# New allergy drug excipient allergy

**DOI:** 10.1186/2045-7022-4-S3-P134

**Published:** 2014-07-18

**Authors:** Vipulkumar Shah

**Affiliations:** 1 Allergy & Asthma Clinic, India

## Background

Because of its rarity, drug excipients allergy is often unsuspected by the doctors. Some times the active drug is blamed and discontinued unnecessarily when the real culprit is drug excipients. This was suspected when patients reported exacerbation of urticaria with anti histaminic which are actually used for treating urticaria. After suspecting drug excipient as the cause of allergy.

## Material

To know actual cause it was decided to do patch testing with common drug excipients. The common excipient selected were Yellow Tartrazine, Titanium Dioxide, Sunset Yellow, Brilliant Blue, Quinolone Yellow, Talcum, and Ponaceue 4R.

## Method

Patch testing material were prepared by using the basic material and suitable vehicle. 24 patients were selected for patch testing. Patch was put on patients back after 48 hrs reading were taken to confirm the skin sensitivities.

## Result

Out of 24 patients 15 patients were positive for Yellow Tartrazine only, 3 patients showed positive to Titanium Dioxide. 2 patients were positive to Quinolone Yellow and Talcum. 2 were positive with Brilliant blue. 3 patients were positive Yellow Tartrazine, Sunsets Yellow and Talcum.

## Conclusion

Drug excipients play an important role in causation is required to be standardised for patch testing. This type of patients should be treated in view of such investigations and preferably treated with drug without excipients.

